# Nurses’ coping with patients’ relatives: Attachment style, burnout, and intentions to leave nursing

**DOI:** 10.1093/eurpub/ckac129.177

**Published:** 2022-10-25

**Authors:** A Gur, N Gur-Yaish, E Sher-Censor, A Zisberg

**Affiliations:** 1 Department of Health Systems Management, The Max Stern Yezreel Valley College, Yezreel Valley, Israel; 2 Faculty of Graduate Studies, Oranim Academic College of Education, Tivon, Israel; 3 School of Psychological Sciences, University of Haifa, Haifa, Israel; 4 The Cheryl Spencer Department of Nursing, University of Haifa, Haifa, Israel

## Abstract

**Background:**

Conflictual interactions with patients’ relatives are prevalent in the work of hospital nurses. These situations may increase burnout and result in intentions to leave the nursing profession and high rates of turnover. It is important to understand the coping mechanisms and behaviours that nurses employ in such conflicts, to help them develop more adequate strategies that could prevent these outcomes. This study aimed at revealing how nurses’ attachment styles colour their behavioural coping mechanisms when dealing with such interactions with patients’ relatives, and how they are related to burnout and intentions to leave the profession.

**Methods:**

An online survey was completed by 140 hospital nurses that included three scenarios of typical conflicts with patients’ relatives. Each scenario was followed by questions that assess stress and inadequacy when handling such situations, and behaviours: problem-solving responses, emotional support seeking, avoidance, and escalating responses. The survey also included self-reports of attachment styles, burnout, and intentions to leave nursing. Data were analysed using SEM (Amos 23).

**Results:**

The model shows an acceptable fit (χ2(24) = 39.33, p = .025; CFI = .963; RMSEA = .068). Higher anxious attachment was associated with higher stress and feelings of inadequacy in handling the situation, which in turn were associated with more emotional support seeking, avoidance, and escalating responses. Escalating responses were indirectly associated with intentions to leave the nursing profession via higher burnout. Higher avoidant attachment was associated with fewer problem-solving responses.

**Conclusions:**

Evaluating nurses’ attachment style, cognitions, and behaviours in conflicts with patients’ relatives is imperative for understanding burnout and intentions to leave nursing. Nursing training should include simulations of conflictual interactions with patients’ relatives to help modify maladaptive coping.

**Key messages:**

• Evaluating nurses’ attachment style, cognitions, and behaviours in conflicts with patients’ relatives is imperative for understanding burnout and intentions to leave nursing.

• Training for nursing staff should consider nurses’ awareness of their attachment style to modify maladaptive cognitions and behaviours in conflicts with patients’ relatives.

